# Low-Actuation Voltage MEMS Digital-to-Analog Converter with Parylene Spring Structures

**DOI:** 10.3390/s150921567

**Published:** 2015-08-28

**Authors:** Cheng-Wen Ma, Fu-Wei Lee, Hsin-Hung Liao, Wen-Cheng Kuo, Yao-Joe Yang

**Affiliations:** 1 Department of Mechanical Engineering, National Taiwan University, Taipei 10617, Taiwan; E-Mails: jeson@mems.me.ntu.edu.tw (C.-W.M.); fuweilee1221@mems.me.ntu.edu.tw (F.-W.L.); ice@mems.me.ntu.edu.tw (H.-H.L.); 2 Department of Mechanical and Automation Engineering and Graduate Institute of Industrial Design, National Kaohsiung First University of Science and Technology, Kaoshiung 824, Taiwan; E-Mail: rkuo@ccms.nkfust.edu.tw

**Keywords:** microelectromechanical systems, digital-to-analog converter, parylene, electrostatic microactuators, white-light interferometry, micromirror

## Abstract

We propose an electrostatically-actuated microelectromechanical digital-to-analog converter (M-DAC) device with low actuation voltage. The spring structures of the silicon-based M-DAC device were monolithically fabricated using parylene-C. Because the Young’s modulus of parylene-C is considerably lower than that of silicon, the electrostatic microactuators in the proposed device require much lower actuation voltages. The actuation voltage of the proposed M-DAC device is approximately 6 V, which is less than one half of the actuation voltages of a previously reported M-DAC equipped with electrostatic microactuators. The measured total displacement of the proposed three-bit M-DAC is nearly 504 nm, and the motion step is approximately 72 nm. Furthermore, we demonstrated that the M-DAC can be employed as a mirror platform with discrete displacement output for a noncontact surface profiling system.

## 1. Introduction

Digital-to-analog converters (DACs), which output electric voltage signals on the basis of binary digital inputs, are widely-used electronic components for numerous applications. During the past few decades, various microdevices that generate mechanical outputs according to binary input signals have been proposed. In general, these devices can be classified into two types based on the outputs. One type of device generates discretized displacements or angles. These devices are called microelectromechanical DACs (M-DACs) [1,2,3,4,5,6,7,8], which are analogous to electrical DACs and can provide repeatable displacements/angles without feedback control circuits. The other type of device generates discretized flow rates or flow concentrations. These devices are called fluidic DAC (F-DACs) [9,10].

In [1], an M-DAC mechanism comprising an N-stage network of connected suspensions was proposed. The mechanism is analogous to the R–2R resistor network for electrical DACs. Sarajlic *et al.* [2] presented a 12-bit M-DAC that was implemented using a novel mechanism with an improved kinematic design that enables both a large output range and high positioning resolution at lower driving voltages. Zhou *et al.* [3] presented a torsional M-DAC mechanism comprising a rigid platform, an array of torsional microactuators and a set of connection beams that connect the actuators to the platform for generating torsional motions. Han *et al.* [4] proposed high-accuracy digital-to-analog actuators with linear load springs that modify the modulation line slope induced by fabrication errors, reducing the influence of fabrication errors on the displacement output. In [5], a four-bit M-DAC integrated with parallel-plate electrostatic actuators was presented. The proposed device contained a movable platform, an array of actuators and a set of connection springs that connect the actuators to the platform. In [6], an open-loop, digitally-driven precision positioning mechanism, which is in fact an out-of-plane MDAC device, was presented. Its application in a grating light modulator was also demonstrated. In [7], an M-DAC driven by thermal actuator arrays was proposed. The device was based on the weighted-stiffness principle, which is similar to that of weighted resistor DACs in electronic circuits. Yeh *et al.* [8] demonstrated in-plane three-bit M-DAC implemented by cascading three lever arm stages and driven by thermal actuator arrays. In [9], a digital microflow controller, which employed a fluidic DAC (F-DAC), was proposed. The proposed F-DAC comprised binary-weighted flow resistance for achieving finer flow-rate levels. Chen *et al.* [10] reported a four-bit F-DAC involving a generalized digitally-switched mixing network that can be used for generating arbitrary dynamic chemical signals. The proposed F-DAC effectively produced discretized chemical concentrations in a constant solvent stream.

For an M-DAC, a set of integrated microactuators are required to generate the displacement outputs. Few of the aforementioned studies on M-DACs have employed the microactuators using the electrothermal effect (*i.e*., thermal actuators). The typical input driving voltages for the thermal actuators may be less than 10 V. However, thermal actuators elevate the overall temperature of M-DACs, which usually leads to noticeable output errors. Therefore, M-DAC devices equipped with thermal actuators have not been demonstrated for practical applications, such as optical communication, RF communication and optical measurement, that require components with high precision positioning. By contrast, M-DAC devices driven by electrostatic microactuators produce higher accuracy. Nevertheless, the typical actuation voltages (*i.e*., pull-in voltages) for electrostatic actuators are approximately 20–60 V [11]. In some instances, the required voltages exceed 100 V [1].

The current study proposes an electrostatically-actuated M-DAC device with a relatively low actuation voltage. The spring structures of the device were monolithically fabricated using parylene-C, whereas other components, such as the actuation electrodes and movable platform, were realized using silicon. Because the Young’s modulus of parylene-C is considerably lower than that of silicon [12], the electrostatic actuators in the proposed device require considerably lower actuation voltages. The M-DAC is driven by a set of out-of-plane electrostatic parallel-plate actuators for actuating the movable platform. The actuation voltages of the actuators predicted by analytical models will also be provided. The fabrication process, which was applied to monolithically implement parylene flexures in the silicon rigid structure, is also described. Furthermore, the measured results, including the transient behaviors of the electrostatic actuator with parylene springs and the actuation voltages of the electrostatic actuators, are presented. In this paper, we also demonstrate that the proposed M-DAC device can be used as a mirror platform for a noncontact surface profiling system. The rest of this paper is organized as follows: Section 2 presents the M-DAC design; Section 3 explains the device fabrication processes; Section 4 contains the measurement results and discussions; and Section 5 presents the conclusion.

## 2. M-DAC Design

**Figure 1:**
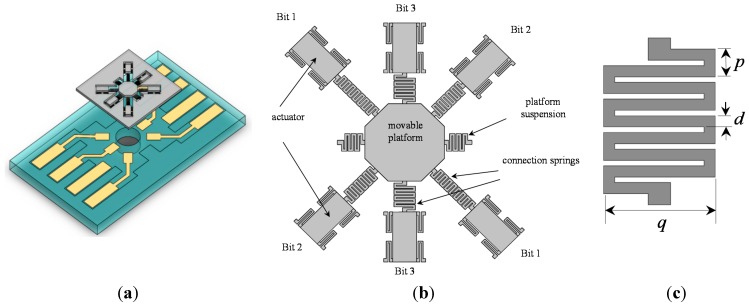
(**a**) Schematic of the microelectromechanical digital-to-analog converter (M-DAC) device; (**b**) top view of the M-DAC layer; (**c**) top view of a connection spring.

**Figure 2:**
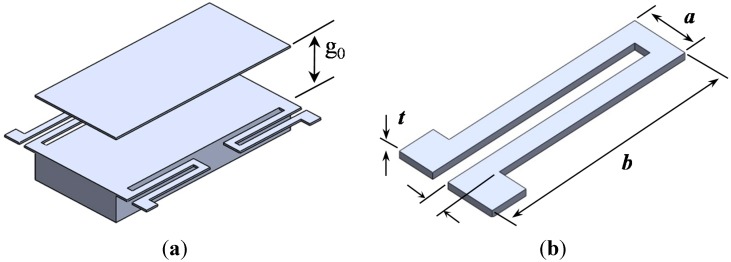
Schematic of the device components: (**a**) schematic of a microactuator; (**b**) schematic of a tether.

Figure 1a shows the schematic of an out-of-plane three-bit M-DAC device. The mechanism is analogous to an electric DAC commonly known as the R–2R resistor network [5]. This device comprises an out-of-plane movable platform, three pairs of parallel-plate capacitive microactuators, three pairs of connection sprin*g*s with binary-weighted stiffness and one pair of platform suspensions. These components are implemented by bonding two layers: the M-DAC layer and the electrode layer (Figure 1b). Section 3 describes the fabrication of these two layers. As shown in Figure 2a, the movable electrode of a microactuator is supported by four tethers. Each microactuator pair, which comprise two microactuators located on opposite sides, represents a “bit” of the M-DAC device. When the bit is “0”, both actuators are off. When the bit is “1,” both actuators’ movable electrodes are pulled down to the fixed electrodes, because the applied voltage is greater than their pull-in voltage. Therefore, the movable platform is also pulled down with a specific displacement. A parylene-C layer was deposited on the surface of the bottom electrode for preventing the two electrodes from directly contacting each other when the microactuator is activated.

The spring constants of the connection springs are specially designed to ensure that the displacement of the movable platform is discretized based on the actuation statuses (*i.e*., input binary codes) of the three pairs of the parallel-plate microactuators. Thus, a displacement output can be generated by combining the input binary bits. The analytical formula of the displacement of the platform involving different combinations of input bits is presented in [5]. 

The minimum voltage required for activating each microactuator is its pull-in voltage ($V_{PI}$), which is expressed as follows [13]:

(1) $$V_{PI}=\sqrt{\frac{8k_{z}{g_{0}}^{3}}{27{\text{ε}}_{0}A}}$$

where $k_{z}$ is the equivalent spring constant of the parallel-plate microactuator, $g_{0}$ is the original gap between the movable and fixed electrodes, ${\text{ε}}_{0}$ is the permittivity of free space and A is the plate area. 

Because $k_{z}$ is the total stiffness of the four tethers supporting the movable electrode plate, it can be written as follows:

(2) $$k_{z}=4\cdot k_{z}^{*}$$

where $k_{z}^{*}$ is the equivalent spring constant of each tether in the *z* direction [14]. The analytical form of $k_{z}^{*}$ is expressed as follows:

(3) $$k_{z}^{*}={\left[\frac{16a^{3}+b^{3}}{6EI_{x}}+\frac{10a^{2}b+ab^{2}}{2GJ}-\frac{a^{2}{\left[\frac{2a}{EI_{x}}+\frac{3b}{GJ}\right]}^{2}}{2\left(\frac{a}{EI_{x}}+\frac{b}{GJ}\right)}\right]}^{-1}$$

where a is the length of the meandering spring in the x direction, b is the length of the meandering spring in the y direction, E is Young’s modulus, G is the shear modulus, $I_{x}$ is the *x*-axis moment of inertia and J is the torsion constant. Figure 2b is the schematic of the spring. Figure 2 shows the design parameters a, b, w and t.

In general, M-DAC devices are fabricated with silicon-based materials. Because silicon is a relatively stiff material, the required operational voltages for electrostatic actuators integrated with M-DACs are approximately 40 V or higher [1,2,5,6]. According to Equation (1), the operational voltage of an M-DAC can be reduced using several methods. For example, we may reduce the gap *g*_0_, increase the plate area *A* or reduce the spring constant *k_z_*. For various precision-positioning applications, *g*_0_ cannot be changed freely, because it affects the DAC displacement outputs, which are strongly dependent on the requirement of the applications. In addition, increasing *A* may not be a favorable approach because of the increase in the size of the DAC chip. 

In this study, we propose using a softer material, parylene-C, to realize the tether and spring structures for the M-DAC, so that the equivalent spring constants of the system can be effectively reduced, ensuring the reduction of the required operational voltage. Table 1 and Table 2 list the parameters used in Equations (1)–(3). 

Table 3 shows a comparison of the pull-in voltages for the microactuators whose tethers comprised a single-crystal silicon and parylene-C. These results were evaluated using typical $g_{0}$ values. The comparison revealed that the pull-in voltage of the device with parylene tethers was approximately one-sixth of that with silicon tethers. Specifically, the pull-in voltage can be effectively reduced by replacing silicon with parylene as the material for the springs.

**Table 1:** Parameters of the suspension spring.

Description	Parameters	Definitions/Values
Length of the spring in *x*-axis	b (μm)	50
Length of the spring in *y*-axis	b (μm)	250
Width of the spring	w (μm)	20
Thickness of the spring	t (μm)	10
Plate area	A (μm^2^)	350,000
Moment of inertia (*x*-axis)	$I_{x}$ (μm^4^)	$wt^{3}/12$
Torsion constant	J (μm^4^)	$0.229wt^{3}$ [15]

**Table 2:** Material property constants of silicon and parylene.

Material	Silicon [16]	Parylene-C [17]
Young’s modulus (E)	130.919 GPa	4 GPa
Shear modulus (G)	79.92 GPa	1.67 GPa

**Table 3:** Equivalent spring constants and pull-in voltages of the parallel-plate microactuators whose tethers comprised single-crystal silicon or parylene-C.

Material	Silicon			Parylene-C		
$k_{z}$ (N/m)	226.5810			6.0986		
$g_{0}$ (μm)	2	3	4	2	3	4
$V_{PI}$ (V)	13.165	24.185	37.235	2.159	3.967	6.108

## 3. Fabrication

### 3.1. Platform Layer

**Figure 3:**
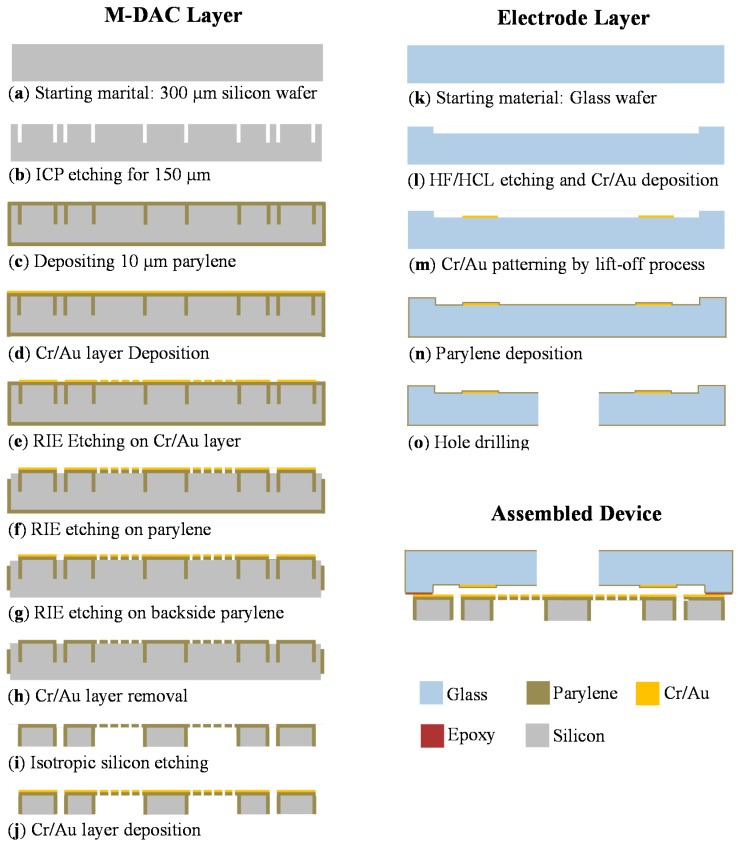
Fabrication process of the M-DAC device.

Figure 3 illustrates the process flow of the M-DAC device fabrication. The device comprises a platform layer and an electrode layer. Figure 3a–h shows the platform layer fabrication process. A silicon wafer (300 µm) served as the starting material. First, the boundary of the platform and the actuators were defined and etched using a inductively coupled plasma (ICP) etching process (20 µm wide and 150 µm deep). Subsequently, a parylene-C layer with a thickness of 10 µm was deposited on a wafer after the A-174 silane adhesion promoter treatment (Specialty Coating Systems, Indianapolis, IN) (Figure 3c) [18]. A dicing saw tape was used to cover the back side of the wafer to avoid parylene coating on the back side. A chromium (200 Å) layer and gold (1600 Å) layer were deposited using an evaporator (Figure 3d). This metal film was patterned using typical lithography with a chromium wet etchant (Sigma-Aldrich) and gold wet etchant (KI:I_2_:H_2_O = 4 g:1 g:40 mL) (Figure 3e). The patterned metal film served as the etching mask for the subsequent parylene etching process. Next, the parylene-C layer was selectively etched using reactive ion etching in oxygen plasma (Figure 3f). The etching reaction governing parylene-C removal was described in [19]. Finally, the silicon wafer was etched using the silicon isotropic etchant, which comprises 126 parts of HNO_3_, 60 parts of H_2_O and five parts of NH_4_F [20]. Because Parylene-C is chemically inert and not attacked by the wet etchant, the portion of the silicon wafer coved by parylene-C is protected during the etching process (Figure 3g). Finally, another Cr/Au layer was deposited on top of the structure for increasing the electrode conductivity and optical reflectivity (Figure 3h). The fabrication process was executed at relatively low temperatures to ensure its compatibility with typical polymer substrates and temperature-sensitive processes. 

### 3.2. Electrode Layer and Device Assembly 

**Figure 4:**
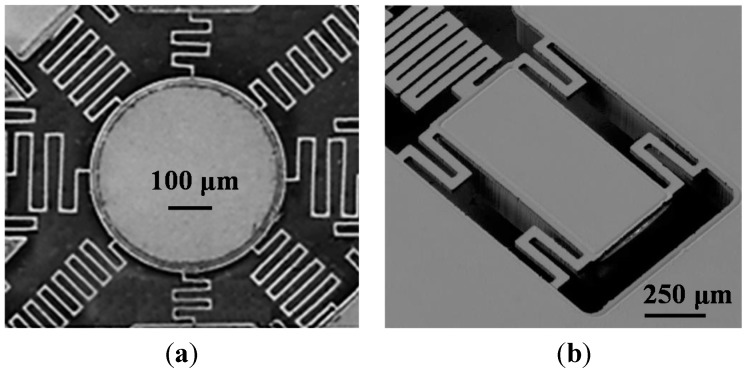
SEM pictures of the fabricated device. (**a**) Movable platform; (**b**) microactuator with four tethers.

**Figure 5:**
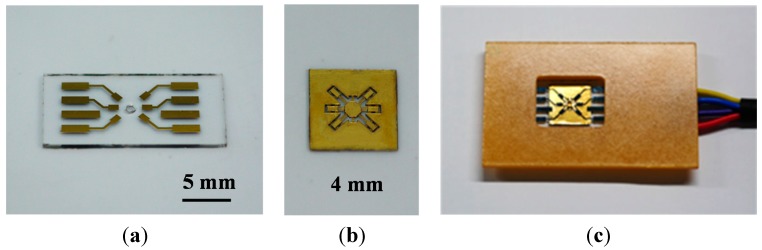
Pictures of the fabricated devices: (**a**) electrode layer; (**b**) M-DAC layer; (**c**) assembled device bonded on a PCB for further testing.

Figure 3k–o illustrates the electrode layer fabrication process. The glass substrate (Pyrex 7740) was etched in hydrofluoric acid and hydrochloric acid (HF/HCl) solution to form a gap spacing, with a photoresist serving as the etching mask (Figure 3k,l). The HF/HCl solution, which was prepared at a volumetric ratio of 10:1 in this study, yields higher surface quality compared to the typical HF etchant [21]. Subsequently, a chromium layer (200 Å) and gold (1600 Å) layer were deposited using electron beam evaporation, and the electrodes were patterned by removing the photoresist (Figure 3m, the lift-off process). A parylene-C layer was deposited as an insulation layer (Figure 3n), and a hole was drilled to expose the surface of the movable platform (Figure 3o). Finally, the platform layer was assembled with the electrode layer (Figure 3p,q). Figure 4a,b depicts the SEM pictures of the movable platform layer and a microactuator. Figure 5a shows the picture of the fabricated electrode layer, and Figure 5b depicts the fabricated M-DAC layer. Figure 5c depicts the assembled device. 

**Figure 6:**
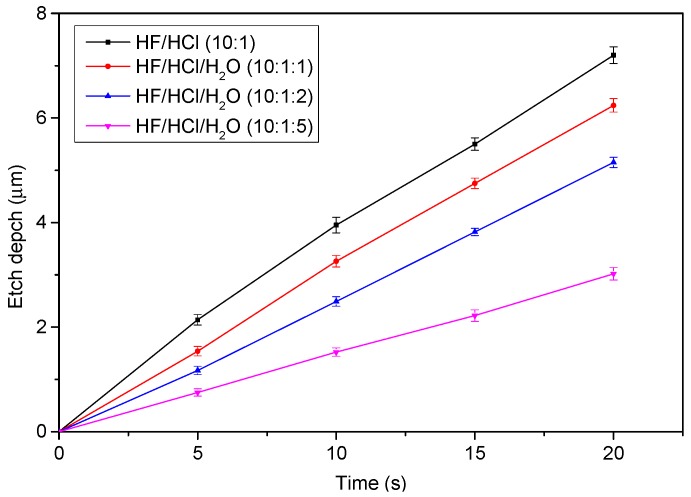
Etching depth *vs*. etching time for various HF/HCl solutions.

Figure 6 illustrates the measured Pyrex glass etching depth *versus* etching time for various HF/HCl solutions (Figure 3l). The etching rate increased with the HF/HCl concentration. Because the depth (*i.e*., $g_{0}$) must be precisely controlled, the etching process was conducted at a slow rate. Although reducing the HF/HCl concentration lowers the etching rate, the etched surface becomes irregular or bumpy if the HF/HCl concentration is too low. In this study, we used an etchant solution with a HF/HCl/H_2_O ratio of 10:1:5.

## 4. Measurement and Discussion

### 4.1. Electrostatic Actuators

**Figure 7:**
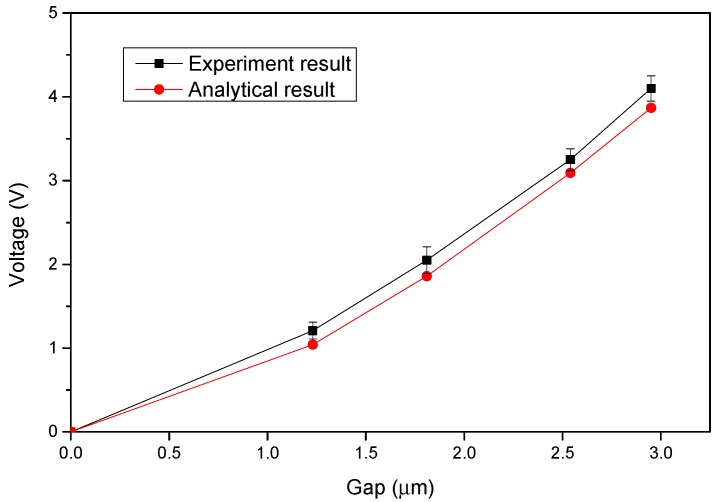
Measured pull-in voltage of the parallel-plate electrostatic actuators with dissimilar gaps.

Figure 7 shows the measured pull-in voltages of the parallel-plate electrostatic actuators with diverse gaps. The voltages were obtained using an LCR meter (Precision Component Analyzer 6440A, Wayne Kerr Electronics Ltd., West Sussex, UK) with DC offset voltages. The DC offset voltage was applied to the microactuator as the actuation voltage and was gradually increased. The capacitance between the two microactuator electrodes was simultaneously measured during the actuation process. The pull-in of the actuator was detected when the measured capacitance suddenly increased. The applied DC offset voltage that induced the pull-in was the corresponding pull-in voltage. Each data point in the figure is the average value of 10 measurements, and the error bars indicate the measured minimum and maximum values. Figure 7 also shows the results predicted by the analytical model (Equation (1)). The total stiffness of the four tethers connected to the movable electrode of the electrostatic actuator was considerably higher than that of the connection spring connected between the electrostatic microactuator and movable platform; therefore, the stiffness of the connection spring is negligible. The measured results were highly consistent with the analytical prediction. 

**Figure 8:**
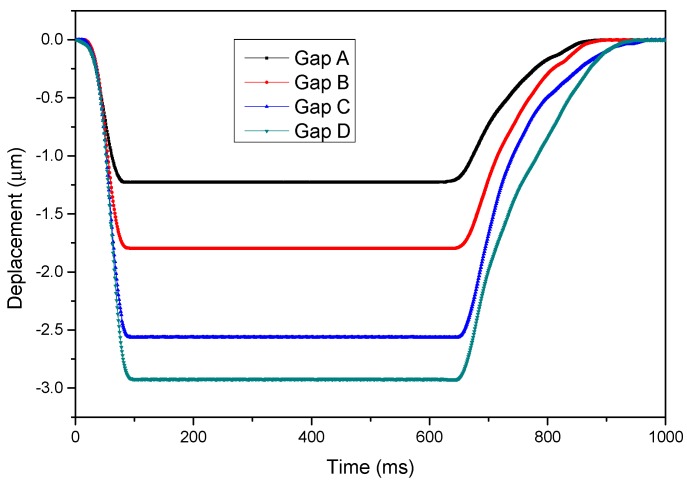
Transient behaviors of the electrostatic microactuator.

The transient out-of-plane motions of the electrostatic microactuator were measured using an interferometer. Figure 8 illustrates the displacements of the movable electrode of the microactuator measured using a laser Doppler vibrometer. Four actuators with dissimilar gaps were measured. For each measurement, a driving voltage of 12 V (which was greater than the pull-in voltages of the actuators for speeding up the dynamics) was applied at *t* = 0. The movable electrode was pulled down to the bottom electrode. When the voltage was released at *t* = 650 ms, the movable electrode returned to its original position. Because the gap between the electrodes was extremely small, the viscous air damping was relatively high. The system was overdamped, and no oscillation was observed. In addition, as shown in Figure 8, when the actuator is actuated, the time required for the movable electrode to touch the fixed electrode is about 100 ms. However, when the voltage is released, it takes about 300 ms for the movable electrode to retreat to its original position. Furthermore, the air damping effect for the movable platform is not as high as the parallel plate actuators. Therefore, the M-DAC time constant is estimated as approximately 0.5 s. Therefore, the maximum conversion rate of the MDAC is approximately two samples per second (sps).

**Figure 9:**
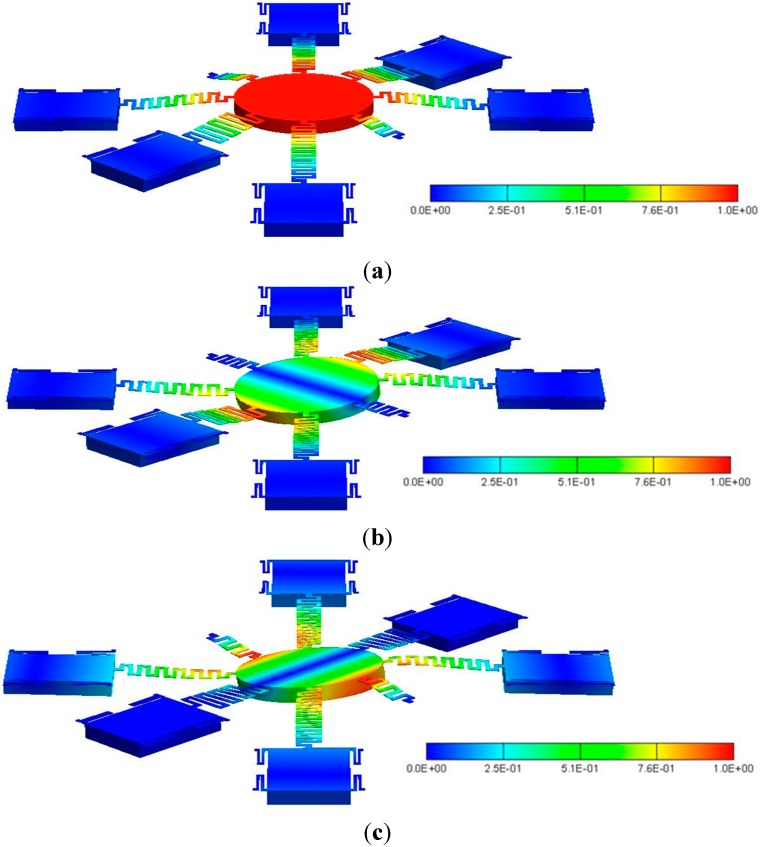
The resonant mode shapes of: (**a**) the first mode; (**b**) the second mode; and (**c**) the third mode.

Table 4 lists the simulated resonant frequencies of the first three modes of the M-DAC device by using Coventorware^®^. The corresponding mode shapes are shown in Figure 9. The first mode complies with the desired motion for the device operation. The second and third modes are the tilting modes. Because of high air damping, the M-DAC device is overdamped. Furthermore, since the parallel-plate actuators are operated in on-off switching mode, the M-DAC device can be considered to be operated in a quasi-static manner. Therefore, these resonant modes will not be induced during operation.

**Table 4:** The resonant frequencies of the first three modes.

Mode No.	1	2	3
Resonant frequencies (Hz)	445	651	1143
Generalized Mass (kg)	2.831 × 10^−7^	6.059 × 10^−8^	7.415 × 10^−8^

### 4.2. Application Demonstration for a White-Light Interferometry System

**Figure 10:**
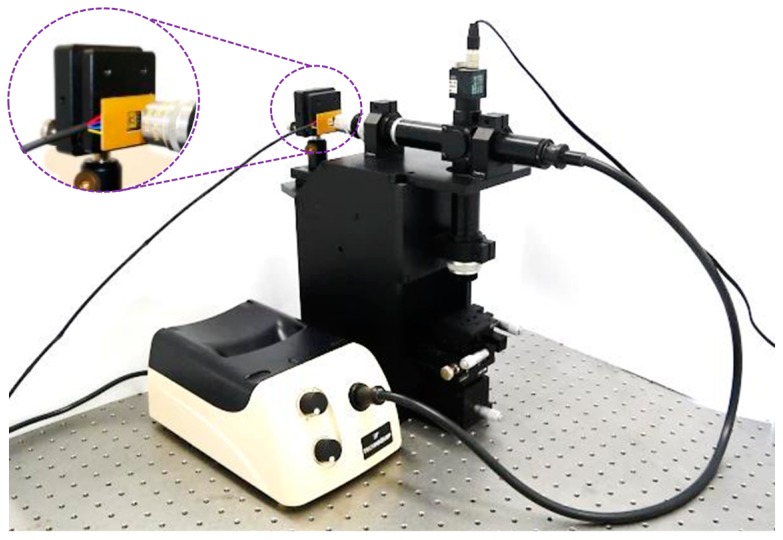
Photograph of the white-light interferometry system.

In this subsection, we demonstrate the application of the M-DAC device in an optical surface profiling system. Figure 10 contains a photo of an optical surface profiling system equipped with the proposed M-DAC device containing parylene springs. The system applies phase-shifting interferometry techniques [22], which require a movable reference mirror whose motion step is ${\text{λ}}_{0}/8$, where ${\text{λ}}_{0}$ is the average wavelength of the photons emitted by the light source (tungsten-halogen lamp). The details of the system are presented in [16]. Because ${\text{λ}}_{0}$ was approximately 570 nm, the motion step of the M-DAC device was designed to be 72 nm. Table 5 lists the design parameters of the meandering springs of the proposed M-DAC device. These parameters are also indicated in the schematic shown in Figure 2c. In addition, $g_{0}$ was designed to be 1.80 µm for the purposes of easy fabrication and low voltage actuation. The spring constants of the springs required for generating the desired motion steps (*i.e*., 72 nm) were evaluated using these parameters and the models described in [16]. Table 5 also lists these calculated spring constants.

**Figure 11:**
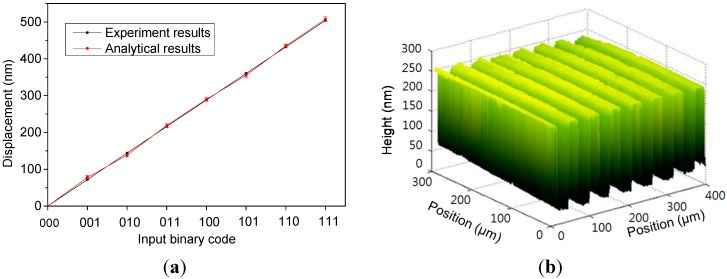
(**a**) Experimental and analytical results of the output displacement of the three-bit M-DAC; (**b**) the shape of the RIE-fabricated grating structure measured using the optical surface profiling system with the M-DAC device.

Figure 11a illustrates the measured displacements of the three-bit M-DAC device as a function of input control codes. The displacements of the movable platform with respect to various digital control codes (*i.e*., from “000”–“111”) were measured using a laser Doppler vibrometer. A 6-V actuation voltage was applied to the electrostatic microactuator. The displacements increased linearly with the input binary codes. Each data point in Figure 11a is the average value of 10 measurements, and the error bars indicate the measured minimum and maximum values. Figure 11b shows the shape of a grating structure measured using the optical surface profiling system. The grating structure was created by etching a silicon substrate with RIE. During the measurement, the interferogram for each phase was acquired by sequentially varying the input binary codes of the M-DAC device from “000”–“110”. The surface profile was obtained by analyzing the interferogram images by using the seven-point algorithm [23]. 

**Table 5:** Parameters of the 3-bit M-DAC.

Description	Bit 1 Springs	Bit 2 Springs	Bit 3 Springs	Platform Suspensions
Length in *x*-axis *p* (μm)	40	40	50	40
Length in *y*-axis *q* (μm)	125	150	205	95
Number of repeated patterns *n*	6	4	2	2
Width *d* (μm)	10	8	8	8
Calculated spring constant *k* (N/m)	0.088	0.173	0.346	1.618

## 5. Conclusions

In this study, we developed a low-actuation-voltage M-DAC device integrated with parylene spring structures. For reducing the actuation voltage, we propose an M-DAC device comprising springs that are fabricated using parylene-C. Because the Young’s modulus of parylene-C is considerably lower than that of silicon, the electrostatic microactuators in the proposed device require considerably lower actuation voltages. The experimental results, including the pull-in voltage of microactuators with different gaps, the transient behaviors of the microactuators and the output displacement of the three-bit M-DAC, are presented in this paper. The typical actuation voltage is 6 V. The measured displacement of the M-DAC device is nearly 504 nm, and the motion step is approximately 72 nm. The application of the proposed M-DAC device is also demonstrated by installing it in an optical surface profiling system.
